# Technological Advancements in Human Navigation for the Visually Impaired: A Systematic Review

**DOI:** 10.3390/s25072213

**Published:** 2025-04-01

**Authors:** Edgar Casanova, Diego Guffanti, Luis Hidalgo

**Affiliations:** Universidad UTE, Av. Mariscal Sucre, Quito 170129, Ecuador; edgar.casanova@ute.edu.ec (E.C.); lahidalgo@ute.edu.ec (L.H.)

**Keywords:** navigation systems, visual impairment, assistive technologies, artificial intelligence (AI)

## Abstract

Visually impaired people face significant obstacles when navigating complex environments. However, recent technological advances have greatly improved the functionality of navigation systems tailored to their needs. The objective of this research is to evaluate the effectiveness and functionality these navigation systems through a comparative analysis of recent technologies. For this purpose, the PRISMA 2020 methodology was used to perform a systematic literature review. After identification and screening, 58 articles published between 2019 and 2024 were selected from three academic databases: Dimensions (26 articles), Web of Science (18 articles), and Scopus (14 articles). Bibliometric analysis demonstrated a growing interest of the research community in the topic, with an average of 4.552 citations per published article. Even with the technological advances that have occurred in recent times, there is still a significant gap in the support systems for people with blindness due to the lack of digital accessibility and the scarcity of adapted support systems. This situation limits the autonomy and inclusion of people with blindness, so the need to continue developing technological and social solutions to ensure equal opportunities and full participation in society is evident. This study emphasizes the great advances with the integration of sensors such as high-precision GPS, ultrasonic sensors, Bluetooth, and various assistance apps for object recognition, obstacle detection, and trajectory generation, as well as haptic systems, which provide tactile information through wearables or actuators and improve spatial awareness. Current navigation algorithms were also identified in the review with methods including obstacle detection, path planning, and trajectory prediction, applied to technologies such as ultrasonic sensors, RGB-D cameras, and LiDAR for indoor navigation, as well as stereo cameras and GPS for outdoor navigation. It was also found that AI systems employ deep learning and neural networks to optimize both navigation accuracy and energy efficiency. Finally, analysis revealed that 79% of the 58 reviewed articles included experimental validation, 87% of which were on haptic systems and 40% on smartphones. These results underscore the importance of experimentation in the development of technologies for the mobility of people with visual impairment.

## 1. Introduction

Navigating complex environments is a daily challenge for people with visual impairments. Although traditional tools such as canes and guide dogs have provided crucial assistance, they have limitations in terms of autonomy and interaction with a complex and dynamic environments. Fortunately, recent technological advances provide new ways for people with visual impairments to perceive their surroundings [1]. These developments have opened up new possibilities for improving the quality of life of people with visual impairment, providing them with greater autonomy and safety in navigating their environment [2]. Moreover, emerging technologies have allowed for the design of more innovative tools and devices that take advantage of advanced sensors and artificial intelligence [3], offering more accurate and effective solutions for navigation. These technologies range from portable sensory devices to advanced computer vision systems [4]. For example, in recent years, the development of smart devices, such as electronic glasses and smart belts, that use technologies such as computer vision, sonar, and real-time positioning systems to provide real-time sensory feedback, have improved the mobility of people with visual impairment and enabled greater social integration [5]. This has reduced physical barriers and offered new opportunities in their daily lives, as well as brought to attention the need to improve the accessibility and personalization of systems, the energy efficiency of devices, and the integration of emerging technologies such as Li-Fi and edge computing. In addition, this highlights that current technologies have not yet been fully explored in diverse and complex environments. Unlike previous studies, this analysis addresses the integration of emerging technologies; furthermore, by focusing on more recent research (2019–2024), it provides an updated view of advances in navigation technology for people with visual impairment, showing how research has evolved and which areas remain critical for future development.

Given the social significance of these technologies, this article aims to provide a systematic review of the latest advancements in navigation for individuals with visual impairments, highlighting how technological innovations are shaping and transforming the field. Our review employs the PRISMA 2020 methodology [6] to consolidate evidence from various studies and analyze their contributions to the field. This review presents a complete investigation on current technologies such as smartphone technologies, systems with artificial intelligence, haptic systems, and navigation algorithms. Fifty-eight articles published between 2019 and 2024 were selected from 39 indexed journals, coming from three databases. The average number of citations per article was 4.55, reflecting a moderate interest in the research topic in the scientific community. These articles were written by a total of 228 authors, indicating a broad collaboration between researchers from different institutions and countries.

The subsequent sections are structured as follows: Section 2 summarizes other key systematic articles that provide an in-depth analysis of technological advances for human navigation in visually impaired people. Section 3 describes the survey methodology, including search formulas, study selection, and inclusion and exclusion criteria. It describes the searched databases and explains how relevant articles were identified, screened, and selected for review. The review results are presented in Section 4, where the current advances in human navigation in visually impaired people are described and categorized in groups. Section 5 discusses the findings of this critical review and the implications in the quality of life of visually impaired people. Finally, Section 6 concludes this review and states new challenges in the development of technologies for human navigation.

## 2. State of the Art

Recent advances in navigation aids for visually impaired individuals focus on wearable devices and electronic travel aids (ETAs) that use technologies such as ultrasonic sensors, LiDAR, and computer vision. Systematic reviews highlight their effectiveness in improving safe mobility while addressing challenges such as power consumption, user comfort, and environmental limitations. This section summarizes key systematic articles that provide an in-depth analysis of technological advancements in navigation aids for visually impaired people.

In the article entitled “Wearable Obstacle Avoidance Electronic Travel Aids for Blind and Visually Impaired Individuals” presented by Xu et al. [7], a systematic review was presented in the IEEE Access journal in 2023. This review contains 89 articles ranging from 2020 to 2023 that focus on portable electronic travel aids (ETAs) designed for people with visual impairments. The review had as main objective the evaluation of the effectiveness and technological advances of these devices in improving the safe navigation of people with visual impairment. The author used the PRISMA guidelines, conducting a search in various databases to identify relevant studies that address the different types of technology used in these devices. The review also analyzed various applied technologies including ultrasonic methods, infrared, laser, and computer vision. Advantages and limitations were presented, such as the dependence on light conditions or difficulties in detecting certain surfaces, considering that they have to operate both indoors and outdoors. The challenges faced by portable Assistive Technology Systems (ATSs), such as the balance between detection accuracy, power consumption, weight, and user comfort, are indicated. The importance of addressing these issues in the design of devices is also highlighted to ensure their effectiveness and long-term use. The review concludes the importance of developing effective wearable ATSs to improve mobility and independence for blind and visually impaired people.

In another article entitled “Survey and analysis of the current status of research in the field of outdoor navigation for the blind” presented by Lian et al. [8], the authors provide a review of 179 articles ranging from 2020 to 2021. This review was published in 2021 and addresses the limitations and advances of technological aids to improve the mobility of blind people in outdoor environments. The review is divided into five key areas: system equipment, highlighting wearable devices as the most common setup, which includes the use of ultrasonic sensors and more advanced technologies such as cameras, LiDAR, and RFID; data sources, where vision sensors based on RGB and RGBD data are most common, demonstrating the increase in the use of computer vision to sense environmental information and detect obstacles; guidance algorithms, which explore the use of localization algorithms, with improvements in accuracy and efficiency; optimization methods, where advances in computer vision are improving the acquisition of information to guide blind individuals; and finally, navigation maps, like tactile and auditory maps that still have limited development and use.

Expanding on this topic, the article “A comprehensive review of navigation systems for visually impaired individuals” [9], published in 2024, reviews 102 articles in the decade between 2014 and 2023. The review presents both traditional aids and electronic devices for navigation. That paper categorizes and evaluates various technological solutions, paying special attention to advances in electronics, sensors, artificial intelligence, and feedback mechanisms. Smartphone and satellite navigation-based systems to improve users’ independence and quality of life are also mentioned. Navigation assistance systems are divided into four categories: visual imaging systems, non-visual data, map-based solutions, and 3D sound. The integration of technologies such as ultrasonic and LiDAR sensors to detect obstacles and provide real-time feedback is also mentioned. A significant evolution in navigation technology is shown, with modern electronic systems showing great effectiveness in detecting obstacles and improving safe mobility. However, it also identifies challenges faced by portable systems, including limitations in power consumption, battery life, and complexity of the estimation algorithms. The benefits of integrating RGB-D sensors with tactile sensors leading to improved perception, object manipulation, and safety are also presented.

In the same vein, in the article “A Qualitative and Quantitative Analysis of Research in Mobility Technologies for Visually Impaired People” [10], an analysis of 140 research studies on assistive technologies for visually impaired people is presented. The review covers a 75-year period (1946–2022). It focuses on the evolution of technological aids, specifically obstacle detection, localization, object recognition, and depth estimation. The results show that aids based on optical and sonic sensors have an average performance of 62%, while those based on more complex technologies such as cloud and artificial intelligence present a lower performance, due to their higher computational needs. Multisensory fusion and robotic aids emerge as the most promising options, with a performance score of 51%. The importance of human acceptance and adaptability is indicated by addressing key challenges such as public transportation and navigation in dynamic environments. The study is based on the increasing prevalence of visual impairment worldwide, making independent mobility difficult. The authors highlight that people with visual impairments encounter environmental obstacles and face an increased risk of falling, leading to increased anxiety, reduced quality of life, and a higher likelihood of depression and other health-related issues.

Finally, expanding on this topic, the study “A Systematic Review of Wearable Devices for Orientation and Mobility of Adults With Visual Impairment and Blindness” [11], presents a systematic review following the PRISMA 2020 guidelines. A search of six academic databases was conducted to identify studies on portable mobility and orientation devices for people with visual impairment. The search focused on adults with low vision or blindness, and 61 articles were selected according to eligibility criteria, including the use of technologies such as ultrasonic sensors and computer vision for the detection and recognition of obstacles. Ultrasonic sensors were the most widely used, demonstrating benefits in mobility performance and in the detection of complex obstacles such as stairs and moving objects. The accuracy in obstacle detection of computer vision-based technologies, such as RGB-D cameras, was also highlighted.

The PRISMA methodology was used rigorously; first duplicates were eliminated using Parsifal Version 1.0. bibliographic management software to identify and eliminate repeated articles automatically and manually. Then, the process of reviewing the articles was performed in two phases: first, an evaluation of the titles and abstracts was made to filter out those that did not meet the inclusion criteria, and then, the selected articles were evaluated in depth by reading them thoroughly, verifying that they met the established requirements. Additionally, for the selection of the articles, parameters such as the relevance of the topic, the most-cited articles, and the innovation of the technology presented were used. All this helps the methodology that ensures that the selection process of the articles is systematic and free of bias.

To conclude, Table 1 presents a summary and a comparison of recently published review articles on navigation technologies for visually impaired people. From this analysis, it can be argued that despite progress, limitations persist, such as limitations in sensor accuracy and complexity in map production. This highlights the need to continue researching and developing more effective solutions to overcome these technological barriers in the field of engineering devices for assistive technologies for the visually impaired. For this reason, the current article updates the review of technological advances in navigation in people with blindness, including smartphone technologies and sensors, haptic systems, mobile devices, and navigation algorithms. This could open up new fields of research in the future and make an important contribution to the welfare of visually impaired people.

## 3. Methodology

### 3.1. Search Strategy

Performing a systematic literature review requires designing the protocol, adjusting the search formulas, filtering the results, selecting articles that meet the inclusion criteria, and removing those articles that meet the exclusion criteria. To initiate this process, a comprehensive literature search was performed using the Scopus, Dimensions, and Web of Science (WoS) databases. The next step was to formulate an article search strategy that fits the purposes of this review. After comparing and analyzing different combinations of search terms for retrieval, the terms “navigation”, “technologies”, “devices”, and “blind people” were chosen as keywords. The keywords were then combined using Boolean operators and adjusted to fit the specific search rules of each database. The search was restricted to articles published between 2019 and 2024, with detailed queries shown in Table 2.

### 3.2. PRISMA Methodology

**Figure 1:**
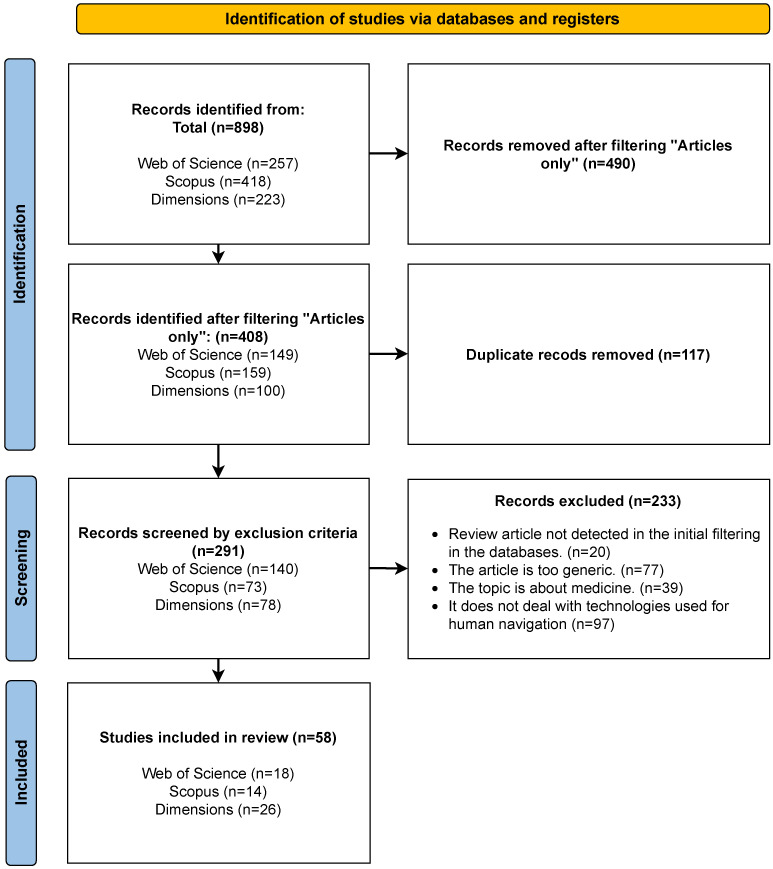
Survey methodology based on the PRISMA 2020 guidelines.

The identification, selection, and inclusion of studies followed the methodology of Preferred Reporting Items for Systematic Reviews and Meta-Analysis 2020 (PRISMA) [6]. The PRISMA methodology applied in this review is illustrated in Figure 1. A total of 898 articles were recovered from the initial search. To refine the results, only documents classified as full articles were included, resulting in 408 articles that were retained for further analysis.

The PRISMA methodology was rigorously used. First, duplicates were eliminated using the Parsifal bibliographic management software to identify and eliminate 117 repeated articles automatically from the database, reducing the total to 291 articles. Then, the process of reviewing the articles was performed in two phases: first an evaluation of the titles and abstracts was made to filter out those that did not meet the inclusion criteria, and then the selected articles were evaluated in depth by reading them carefully, verifying that they met the established requirements (Table 3). The selection criteria focused on identifying articles exploring advanced technologies for human navigation, with a particular focus on the visually impaired. A total of 58 articles remained after this screening. These 58 articles were included in the current review.

### 3.3. Bibliometric Analysis

**Figure 2:**
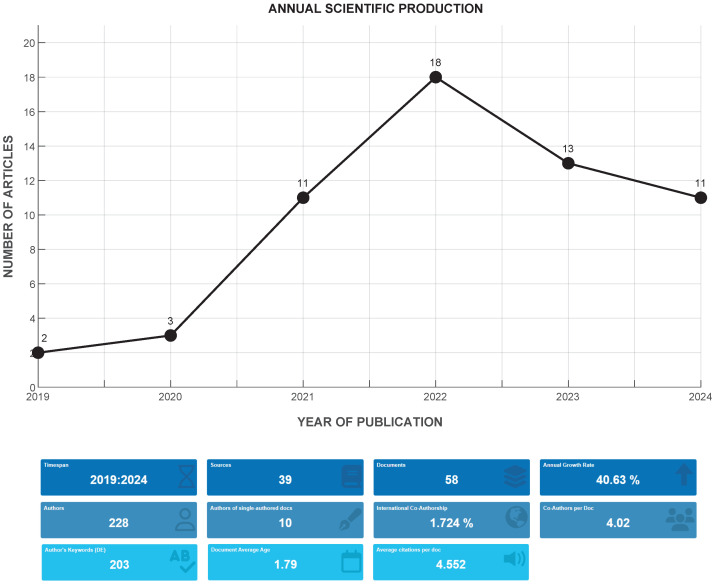
Scientific production in the reviewed database. The upper part shows the production per year in the period 2019 to 2024. The lower part shows relevant details regarding authors, citations, and sources of information, among others. These data are of interest to understand the analyzed database.

A bibliometric analysis of these articles was performed using the Bibliometrix package in RStudio Version 2024.04.2. As can be analyzed in the report presented in Figure 2, the 58 articles belong to 39 indexed journals. Scientific production in the investigated thematic area has been increasing, with the highest research output recorded in 2022 with 18 published articles. The average number of citations per article was 4.55, reflecting moderate interest in the research topic in the scientific community. These articles were written by a total of 228 authors, indicating a broad collaboration between researchers from different institutions and countries (1.724% of international co-authorship).

**Figure 3:**
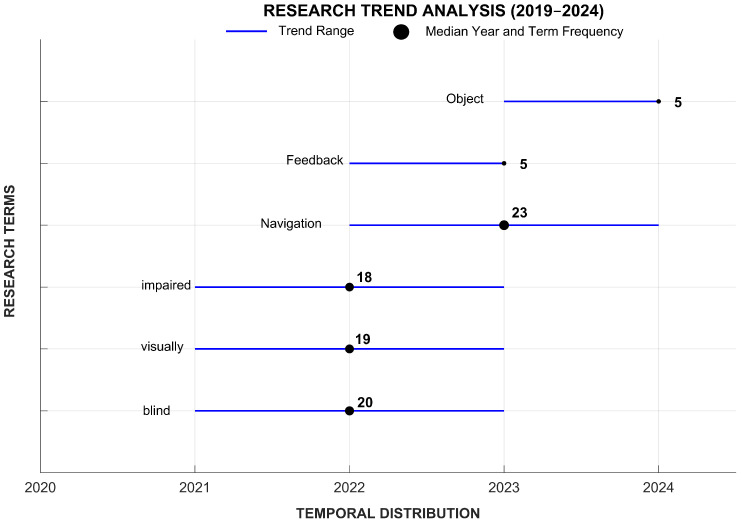
Research trends in the reviewed topic. The frequency of research terms is highlighted with a black marker placed at the median of the year of occurrence. The blue line represents the trend range.

Figure 3 presents a graphical representation of research trends in technological advancements for visually impaired navigation from 2019 to 2024. The analysis reveals a steady increase in the prominence of key terms such as “navigation”, “blind”, “visually”, and “impaired”, indicating a sustained focus on developing assistive technologies. The term “navigation” exhibits the highest frequency (with a term frequency of 23), peaking around 2023, highlighting its central role in recent research. Additionally, emerging terms like “feedback” and “object” appear in later years, suggesting a growing interest in enhancing user interaction and obstacle detection mechanisms. The distribution of terms over time suggests a shift from general assistive concepts toward more specialized technological solutions. These findings underscore the evolving nature of research in this field, reflecting an increasing emphasis on intelligent navigation systems and sensory feedback mechanisms for visually impaired individuals.

**Figure 4:**
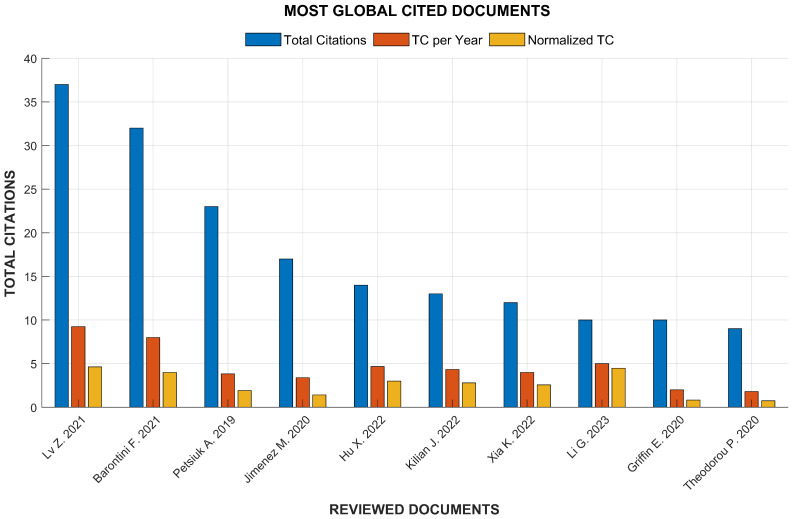
Citation impact on the ten most-cited papers in the analyzed database. Three bars are shown for each document: Total Citations (in blue), Citations per year (in orange), and the Normalized Citation index that accounts for variations in age and field of publication (in yellow). The information was generated with the Bibliometrix package in RStudio. The referenced papers are: Lv Z. [12], Barontini F. [13], Petsiuk A. [14], Jimenez M. [15], Hu X. [16], Kilian J. [17], Xia K. [18], Li G. [19], Griffin E. [20], Theodorou P. [21].

The bibliometric analysis also allowed us to identify the top 10 most-cited documents in the screened database. These articles have the highest impact on citations in the research area, establishing relevant trends, theories, and technologies for human navigation. As shown in Figure 4, the study presented by Lv Zhiqiang et al. [12] presented 37 total citations, 9.25 total citations per year, and a normalized total citation index of 4.63. The total number of citations and the number of citations per year are useful impact metrics, but more relevant is the normalized total citation index. This is a metric that eliminates the bias caused by more recent publications that normally have less time to accumulate citations, and the differences in citation density between different disciplines. For the study presented by Lv Zhiqiang et al. [12], the value of 4.63 for the normalized total citation index means that it has been cited 4.63 times the average number of citations expected for articles published in the same year and research area, which highlights the impact of this article. The rigorous use of Parsifal and bibliometric analysis for the study strengthens the quality and objectivity of the study selection process and allows a more complete evaluation of the evidence and impact of the studies, which enriches the interpretation of the results and increases the reliability of the conclusions.

## 4. Results

The 58 articles in this study were reviewed from their respective abstracts and therefore classified into four distinct groups according to the type of technology and focus on improving the mobility and autonomy of people with visual impairment. The first group focused on systems that use advanced smartphone technologies, with the integration of GPS, ultrasonic sensors, and assistive software to provide real-time navigation support; the second group includes haptic systems, which stand out for their ability to provide tactile feedback, using ultrasonic sensors and artificial vision in devices such as smart canes; the third group encompasses computational algorithms to enhance obstacle detection, localization, and environmental perception; and finally, the fourth group focuses on advances in automatic guidance, combining localization, simultaneous mapping, and object detection through deep learning, optimizing navigation accuracy and energy efficiency.

### 4.1. Smartphone Technologies

In this section, the articles focus on the integration of smartphone-based systems that aim to enhance navigation for people with visual impairments. It includes systems that integrate high-precision GPS, ultrasonic sensors, Bluetooth, and various assistive apps to provide real-time navigation assistance for object recognition, obstacle detection, and path generation. Table 4 presents a summary of key studies within this group, highlighting smartphone integration and experimental validation.

### 4.2. Haptic Systems

Haptic systems were classified by their ability to provide tactile feedback, especially in devices for the visually impaired, such as smart canes that use ultrasonic sensors and machine vision to detect obstacles and transmit information through vibrations or audible alerts. Table 5 presents a summary of key studies within this group, highlighting their experimental validation in real or simulated environments.

### 4.3. Navigation Algorithms in Systems for the Blind

The development of assistive navigation systems for visually impaired people has integrated a wide range of computational algorithms. These algorithms include obstacle detection, path planning, and trajectory prediction, applied to technologies such as ultrasonic sensors, RGB-D cameras, and LiDAR for indoor navigation, as well as stereo cameras and GPS for outdoor navigation. Table 6 presents the main algorithms used in the state of the art, highlighting their applications in different systems and their experimental validation in real or simulated environments.

### 4.4. Systems with Artificial Intelligence (AI)

This group highlights systems that incorporate Artificial Intelligence (AI) to improve navigation for the visually impaired, offering an efficient solution for the navigation with optimized energy consumption, optimizing both navigation accuracy and energy efficiency. One of the outstanding systems is You Only Look Once (YOLO), which is a computer vision technique for real-time object detection. YOLO is an algorithm based on convolutional neural networks (CNNs) that is known for its high speed and accuracy. Unlike other methods that require separate object localization and classification processes, YOLO does this all at once, making it much more efficient and suitable for real-time applications. Table 7 presents a summary of key studies within this group.

Of the 58 reviewed articles, 79% (46 articles) include an experimental phase, demonstrating a high degree of practical validation of the proposed technologies. In particular, 40% of the systems that employ smartphone-based technologies (24 articles) have been experimentally evaluated with real users, highlighting the effectiveness and applicability of these devices in navigation scenarios. Furthermore, 87% of the articles on haptic systems (13 of 15) include experimental evidence, highlighting the applicability and reliability of haptic devices as solutions for the mobility of visually impaired people. Finally, the two articles grouped under the use of artificial intelligence also include an experimental phase.

## 5. Discussion

In recent years, various technological advancements have significantly contributed to improving the navigation capabilities of visually impaired individuals. These innovations, ranging from smartphone-based applications to haptic feedback systems, as well as artificial intelligence and advanced navigation algorithms, have shown great promise in providing more autonomy and safety. This section discusses key advances, highlights limitations of current technologies, and explores potential improvements and emerging trends in the field of assistive navigation for visually impaired people.

Smartphone technologies have played a key role in improving the independence of visually impaired people. A key example is the BlindRouteVision application [22], which uses GPS and ultrasonic sensors for outdoor navigation. Although the application has been well received in terms of usability and functionality, limitations in areas with poor GPS signal remain a significant barrier to its effectiveness in certain environments. The use of Light Fidelity (Li-Fi) technology as an alternative to GPS could offer greater reliability in indoor environments. As demonstrated in the BLi-Fi system as indicated by Siddhi et al. [51], Light Fidelity (Li-Fi) technology, due to its high-speed data transmission capability, superior bandwidth, and reliability, proposes a prototype of the system for indoor navigation for the blind. The proposed idea is to develop a system that improves the ease of indoor navigation for the blind and this navigation technology uses visible light communication. The indoor location information, such as room, hallway, kitchen, etc., is embedded in the light beams transmitted by the LED and the system is integrated with a microcontroller that has a visible light receiver on a stick that collects the data, demodulates it, and communicates it through audio with the actual location to the visually impaired person. Although smartphones and tactile feedback canes offer good levels of accuracy and advantages, their accuracy depends on the use of specific applications or tactile feedback. The nature of these devices are different, but can be complementary in their use.

The use of haptic feedback has proven to be effective in alerting users about immediate obstacles, as indicated by Mohan et al. When using the EyeCane [60], a device designed to help visually impaired people move around their environment using image processing and the Internet of Things, obstacles are identified by the camera and ultrasonic sensor of the smart cane, which also calculates the distance from the user to the object. When an obstacle is detected, a sound is emitted in the user’s headphones to alert him; the cane has a camera that takes images of the environment. These images are analyzed by image processing algorithms to find barriers and other important details. The cane is also connected to the Internet of Things (IoT), allowing it to interact with other devices and offer more functions. In their study, Yerkewar et al. [27] propose a solution of a smart cane with an ultrasonic sensor to detect stairs or a pair of ultrasonic sensors to detect any other obstacle in front of the user, in a range of 1.0–1.5 m. In addition, a humidity sensor is placed in the center of the stick to detect water and puddles, which is especially useful in the rainy season. When an obstacle is detected, a buzzer sounds. This proposed system uses an Arduino nano-microcontroller-embedded system, buzzer, GSM, GPS, IC encoder, IC decoder, and a PCB. This smart blind cane is low-cost, has a fast response, was easy to design, and is lightweight.

Regarding the use of navigation algorithms for blind people, the use of Simultaneous Localization and Mapping (SLAM) has proven to be one of the most promising solutions. This approach allows for real-time mapping and precise localization in complex environments. However, the implementation of SLAM on portable devices still faces significant challenges in real-time processing and adaptation to dynamic environments. Integrating SLAM with artificial intelligence could improve the accuracy and adaptability of the system, but high resource consumption remains an important limitation in small and affordable devices [56]. Combining SLAM with sensing technologies such as depth sensors or smart cameras can also improve environmental perception, facilitating more autonomous and accurate navigation.

The use of artificial intelligence (AI) was also identified in this review as a potential tool for blind navigation. In this context, AI techniques such as YOLO have been integrated to provide real-time object detection. YOLO is a deep learning algorithm that can identify and classify objects rapidly, making it highly efficient in dynamic environments where immediate obstacle recognition is required [63]. AI has significantly improved the accuracy of obstacle detection and route planning. The VISION device [28], for example, uses mobile edge computing and 5G connectivity to process high-resolution camera data in real time, enabling precise object detection and obstacle avoidance. This type of technology, which does not rely on remote servers, reduces latency and improves processing speed. However, high computational demands and energy consumption remain barriers to the widespread adoption of devices that integrate AI. As hardware advances in the form of more efficient processors and optimized machine learning platforms, these systems will become more accessible to end users.

With respect to people’s acceptance of technologies for people with blindness as mentioned by Madake et al. [10] in their article, the majority of these devices and prototypes have been beneficial to serve navigation assistance to some extent. Also, there are some demerits, such as low acceptance, high cost, difficulty to use, ergonomics, and not being easy to carry. Also, Barontini et al. [13] states that despite the promising research outcomes, these solutions have met scarce acceptance in real-world scenarios. Often, this is also due to the limited involvement of real end-users in the conceptual and design phases.

The review articles mentioned in the state of the art introduction in this paper have also identified significant advances, such as the integration of computer vision and AI in portable devices to improve blind navigation. However, a recurring challenge in the literature is the lack of personalization in these technologies, which limits their effectiveness in different contexts and for diverse users. For example, the integration of technologies such as virtual reality and augmented reality in navigation is being explored as a possible solution to improve user interaction with their environment [61]. Furthermore, the use of mobile platforms and 5G connectivity in systems like VISION has opened up new possibilities to improve object detection accuracy and route planning [29]. However, accessibility, cost, and energy efficiency remain critical areas that need more innovation.

Finally, in the context of this research, the most-cited article was the study presented by Lv Zhiqiang et al. [12], who describe a crucial advance in navigation technology for blind people, highlighting the importance of GPSs and other outdoor navigation tools. The authors also propose an indoor algorithm that avoids obstacles and predicts trajectories with high accuracy by focusing on the unique motion characteristics of blind people. This spatiotemporal model demonstrates remarkable improvements in navigation in indoor spaces, such as multi-story shopping malls.

In conclusion, although significant advances have been made in assistive navigation for visually impaired people, current technological limitations suggest the need for greater integration of smart devices with more robust connectivity and more efficient processing systems. Future research should focus on improving accessibility, reducing energy consumption, and integrating emerging technologies such as Li-Fi and edge computing, which could provide more efficient and personalized solutions for visually impaired individuals.

### Limitations and Recommendations

In this research, it was not considered necessary to investigate other databases such as IEEE or PubMed, among others, because the Web of Science (WoS) covers a broad spectrum of scientific research relevant to the topics of importance. WoS is recognized for its exhaustive compilation of the scientific literature, including articles indexed in the IEEE Explore database, which guarantees that the most important articles with the greatest impact on the discipline are included.

The use of other databases could prove valuable in future research, as these platforms offer access to a different set of publications and approaches that could complement the findings obtained, broaden the perspective, and enrich the analysis, especially in more specific or technical areas.

## 6. Conclusions

This research on technological advances in human navigation for visually impaired people allows us to highlight the significant advances made in the development of navigation technologies aimed at improving mobility and autonomy for visually impaired people. In this study, the PRISMA 2020 methodology was applied to perform a systematic literature review of 58 papers published between 2019 and 2024, selected from Dimensions, Web of Science, and Scopus databases. The bibliometric analysis demonstrated a growing interest of the research community in the topic, with an average of 4.552 citations per article and 1.724% of international co-authorship. This research identified four main approaches in navigation technologies: smartphone technologies, haptic systems, navigation algorithms, and AI-based systems. Current technological limitations suggest the need for greater integration of smart devices with more robust connectivity and more efficient processing systems. Challenges for future research should focus on improving accessibility through the development of customizable interfaces and multimodal technologies adapted to different levels of visual impairment, as well as finding ways to optimize costs to make them easily accessible to all; this goes hand in hand with generating energy efficiency in navigation systems. We observed the integration of emerging technologies such as Li-Fi, which could improve indoor navigation with greater accuracy and reliability, and edge computing, which would allow local data processing to reduce latency and increase the autonomy of devices. Future work should perform tests in real environments.

Finally, of the 58 articles reviewed, 79% included experimental validation, 87% of which were on haptic systems and 40% on smartphones. These results underscore the importance of experimentation in the development of technologies for the mobility of people with visual impairment.

## Figures and Tables

**Table 1:** Summary and differences of recently published review articles on navigation technologies for the visually impaired.

Citation	Year of Review	Technologies Reviewed	Publication Year Range of Studied Articles	Number of Studies Included	Type of Navigation	Differences with Respect to Our Paper
[7]	2023	Ultrasonic sensors, infrared, laser, computer vision	2020–2023	89	Wearable	Our study includes more recent papers (2019–2024) and focuses on newer advancements in sensors and algorithms.
[8]	2021	Ultrasonic sensors, cameras, LiDAR, RFID, RGB, RGBD, computer vision	2020–2021	179	Outdoor	Our study includes papers up to 2024, highlighting the latest advancements in computer vision and mobile devices.
[9]	2024	Ultrasonic sensors, LiDAR, artificial intelligence, RGB-D sensors, tactile feedback	2014–2023	102	General	Our review updates technological advancements with a focus on the latest research and developments in AI and mobile navigation systems.
[10]	2022	Optical and sonic sensors, cloud computing, AI, multisensory fusion, robotics	1946–2022	140	Mobility	We provide a more recent and focused analysis of technological advances, emphasizing current sensor integration and the latest AI developments.
[11]	2021	Ultrasonic sensors, computer vision, RGB-D cameras	2010–2020	61	Portable	Our paper includes recent studies from 2019 to 2024 and provides a broader perspective on the integration of newer sensors and navigation algorithms.

**Table 2:** Search formulas used in each database. The asterisk (*) is used to search for word variations.

Database	Search Formula
Scopus	TITLE-ABS-KEY (“navigation*”) AND (TITLE-ABS-KEY (“technolog*”) OR TITLE-ABS-KEY (“device*”)) AND TITLE-ABS-KEY (“blind*”) AND PUBYEAR > 2019 AND PUBYEAR < 2025 AND (LIMIT-TO (DOCTYPE, “ar”))
Web of Science	TS = (“navigation*”) AND TS = (“technolog*” OR “device*”) AND TS = (“blind*”) AND PY = (2020–2024) AND DT = (Article)
Dimensions	(“navigation”) AND (“technologies” OR “devices”) AND (“blind people”)

**Table 3:** Inclusion and exclusion criteria.

Criteria	Details
Inclusion Criteria	- Articles describing navigation technologies (e.g., GPS systems, assistive devices, portable technologies, etc.)
	- The topic is focused on mobility technologies for blind people.
Exclusion Criteria	- Review articles not identified during initial database filtering.
	- Articles with a structure that is too general or lacks specific details.
	- Studies focused on medicine, unrelated to technological navigation systems.
	- Articles that do not address technologies for human navigation.

**Table 4:** Smartphone technologies for the navigation of visually impaired people.

Citation	Main Topic	Technology Used	Smartphone Integration	Does the System Have an Experimental Phase?
[22]	Usability and UX Evaluation of BlindRouteVision App	Android app, high-precision GPS, ultrasonic sensor	Smartphone app for navigation, emergency alerts	Yes, with 30 visually impaired users in a pilot study
[23]	Ultrasonic Navigation Assistance	Ultrasonic transceivers, water sensor, GPS, Bluetooth	Android phone with Bluetooth voice feedback	Yes, in both obstacle and fixed-route modes
[24]	Assistive Technology for Education	Computer and smartphone interfaces	Smartphone apps for user experience improvement	No experimental phase reported
[25]	IoT-Based Quality of Life Enhancement for VI People	Arduino UNO, ultrasonic and proximity sensors	Android app with IoT integration	Yes, tested for efficiency with IoT and apps
[26]	EchoSee for Real-Time 3D Environment Navigation	3D scanning, spatialized audio, real-time updates	Mobile device generating 3D soundscapes	Yes, feasibility study with user testing
[27]	Smart Stick for Obstacle Detection	Ultrasonic sensors, GSM, GPS, Arduino	Not directly smartphone-integrated but GSM notifications sent	No direct experimental phase mentioned
[4]	Navigation App Using NTRIP Protocol	GNSS, RTCM corrections, Android Studio	Screen reader and early obstacle alerts	Yes, tested using NSSDA for precision comparison
[28]	5G Edge Computing for Object Detection in Wearables (VIS4ION)	High-res cameras, 5G networks, wearable device	Smartphone-connected processing via 5G	Yes, simulation-based study with navigation routes
[29]	Smart Blind Walking Stick	Ultrasonic sensor, GPS, audio feedback	Integration through live GPS location	No experimental phase detailed
[30]	GPS and Wearable Tactile Display for Navigation	Smartphone GPS, tactile feedback device in shoes	GPS-based positioning with haptic instructions	Yes, with two user experiments
[18]	Intelligent Blind Guidance System (IBGS)	WiFi, cloud database, speech recognition (ConvT-T)	Not reliant on smartphones; cloud-connected	Yes, with outdoor and speech recognition tests
[31]	IoT-Based Blind Smart Stick	Ultrasonic sensors, SPO2 sensor, Node MCU	GPS-enabled location alerts with user-defined audio input	No detailed experimental phase
[17]	Unfolding Space Glove for Spatial Exploration	Haptic feedback, sensory substitution	Not reliant on smartphones	Yes, with structured training sessions and testing
[21]	Training Framework for Assistive App Acceptance	Interviews, training methodologies	Training-focused smartphone app development	No formal experimental results

**Table 5:** Haptic systems for the navigation of visually impaired people.

Citation	Main Topic	Technology Used	Type of Haptic System	Does the System Have an Experimental Phase?
[32]	Programmable tactile feedback system for blindness assistance	Triboelectric nanogenerator (TENG), self-excited electrostatic actuator (SEEA)	Vibrotactile system with programmable matrix for navigation and Braille assistance.	Yes, evaluated with various applications including Braille and haptic navigation systems.
[33]	A white cane modified with ultrasonic detectors	Ultrasonic sensors integrated into a white cane	Vibrotactile and auditory feedback for obstacle detection at head and waist levels.	Yes, tested with 10 blindfolded participants across three obstacle stations.
[34]	Hand-held haptic navigation devices for current walking	GPS for outdoor and various tracking systems for indoor navigation	Portable vibrotactile devices for indoor and outdoor navigation.	Yes, high success rates reported, though training was limited.
[35]	Navigating the unseen: Ultrasonic technology for blind navigation	Ultrasonic sensors, microcontroller, and buzzer	Vibrotactile and auditory feedback for obstacle detection and object localization.	Yes, demonstrated through scenarios including cane localization and obstacle avoidance.
[1]	Smart Stick for Visually Impaired on Streets Using Arduino UNO	Arduino UNO, ultrasonic sensors, buzzer	Vibrotactile and auditory system providing artificial vision and obstacle alerts.	Yes, focused on real-time obstacle detection and navigation for urban scenarios.
[36]	Smart Glasses for Blind People	Ultrasonic sensors, Raspberry Pi, vibrators, and audio output	Mixed audio and vibrotactile feedback for navigation and obstacle avoidance.	Yes, tested with real-world obstacle avoidance tasks.
[37]	Navigation system for blind people using LiDAR	LiDAR, microcontroller, vibration motor, and buzzer	Vibrotactile and auditory system for real-time obstacle detection.	Yes, verified through field tests for usability and efficiency.
[38]	Smart Glove for Blind	Ultrasonic sensors, GPS, color sensor, and vibration motor	Glove-based vibrotactile feedback with multiple sensors for enhanced mobility.	Yes, tested for obstacle detection, color identification, and fall alerts.
[39]	Real-time wearable navigation support system for BVIP	Fuzzy logic decision-making, Raspberry Pi4, and haptic voice interface	Vibrotactile signals for safe navigation, enhanced with human safety evaluations.	Yes, validated through tests in different environments with visually impaired participants.
[40]	Thermal navigation for blind people	GPS, infrared sensor array, and vibration bracelet	Vibrotactile feedback for thermal-based navigation and orientation.	Yes, tested for precise indoor navigation and orientation.
[41]	Sonar Glass	Sonar sensors, log-polar mapping, and SIFT algorithm	Vibrotactile and auditory alerts for spatial awareness using synchronized visual-like perception.	Yes, simulations and real-world tests conducted in indoor and outdoor environments.
[42]	Smart Shoe for visually impaired	Piezoelectric materials, obstacle sensors, and SOS button	Vibrotactile feedback integrated into footwear for enhanced safety and navigation.	Yes, tested for obstacle detection and emergency assistance.
[43]	AI-equipped assistive devices for blind individuals	Internet of Things (IoT), AI-based object detection, and GPS	Vibrotactile and auditory feedback for integrated obstacle detection and navigation.	Yes, tested for cost-efficiency and multi-functional use.
[20]	Audio–tactile map for visually impaired individuals	Interactive audio–tactile mapping system	Haptic map with mixed vibrotactile and audio feedback for cognitive spatial mapping.	Yes, evaluated with 14 participants for spatial recall and navigation learning.
[44]	Enhanced YOLOv8 with OpenCV for object detection	YOLOv8 object detection, OpenCV for distance measurement	Haptic-enhanced detection for precise obstacle awareness and feedback.	Yes, achieved 3.15% error rate with efficient response times.
[45]	Smart cane with EMF detector for the blind	Ultrasonic sensors, EMF detector, and vibration motor	Vibrotactile and auditory feedback for static and dynamic obstacle detection.	Yes, validated with optimized operational results.
[46]	Vibrotactile belt for challenging situations	Magnetic north-based vibrotactile orientation	Belt with vibrotactile stimuli for spatial awareness and emotional comfort.	Yes, tested over 7 weeks with blind users in various outdoor situations.
[15]	Assistive locomotion device for the visually impaired	Smart walker with haptic controllers	Vibrotactile guidance through physical interaction with the walker.	Yes, tested with blindfolded and sighted participants.
[13]	Wearable haptics for indoor navigation	RGB-D camera, haptic portable device, and force feedback	Haptic wearable devices for force-guided indoor navigation.	Yes, tested with visually impaired and blindfolded participants.
[19]	Cognitive systems for visually impaired individuals	RGB-D camera, embedded computer, and haptic modules	Wearable vibrotactile system with modular task support.	Yes, pilot study evaluated navigation and object recognition tasks.
[14]	Open-source haptic navigation system	Ultrasound-based haptic feedback with vibration patterns	Low-cost vibrotactile system for accessible navigation.	Yes, effective in real-world navigation tasks.
[47]	Cobot tactile display for virtual diagram exploration	Omnidirectional robot base, optical mouse sensor, and admittance control	Haptic robotic assistance for tactile diagram exploration.	Yes, found intuitive and useful by visually impaired participants.
[48]	Pedestrian lane detection for vision-impaired individuals	Deep learning methods for lane detection	Vibrotactile and visual feedback for safe pedestrian lane guidance.	Yes, evaluated on a large dataset of pedestrian lane images.
[49]	VISTATM: Touch aid for sightless navigation	Ultrasonic range detection and vibrotactile feedback	Vibrotactile belt providing proportional distance feedback.	Yes, validated in real-world navigation scenarios.

**Table 6:** Navigation algorithms in systems for the navigation of visually impaired people.

Citation	Main Topic	Technology Used	Algorithm/Method Used	Does the System Have an Experimental Phase?
[50]	SDGs and Visual Impairment	Smartphone cameras, laser rangefinders, Wi-Fi, BLE beacons, smart lighting, barometric sensors, and magnetic fields.	Obstacle detection	No
[51]	BLi-Fi Navigation	Li-Fi, LEDs as transmitters, LDRs as receivers, microcontrollers.	Obstacle detection	No
[52]	Smart Cane for the Elderly and Visually Impaired	Ultrasonic sensors, IoT, vibration motor, buzzer.	Obstacle detection	Yes, tested with elderly users to detect obstacles and provide feedback.
[53]	Sound-Based Prototype for Localization	Ultrasonic sensor networks, GPS, digital compass.	Dijkstra’s algorithm, LSTM	Yes, 45 indoor and outdoor tests with navigation accuracy measurements.
[54]	Safe Navigation with Arduino and 1Sheeld	Arduino Uno, 1Sheeld, ultrasonic and water sensors, buzzer, vibration motor, mini solar panel.	Navigation through obstacle detection	Yes, tested with volunteers to verify navigation safety and system accuracy.
[55]	Trinal Optics	Arduino Uno, camera, Bluetooth headsets, ultrasonic sensors, voice assistant.	Object detection and environment analysis	No
[16]	StereoPilot	RGB-D camera, spatial audio representation (SAR).	Fitts’ Law experiments	Yes, compared spatial navigation performance with SAR and other auditory feedback methods.
[56]	Systematic Review of SLAM	SLAM, various sensors (not specified), machine learning.	Various SLAM-based approaches	No
[57]	Smart Glove for Navigation	Ultrasonic sensors, GPS, vibrators, microcontrollers (PIC).	Environmental data processing	No
[2]	Web Content Accessibility Guidelines (WCAG) for Eye Tracking Navigation	Eye-tracking technology, web navigation.	Quadtree-based goal selection, hierarchical menu representation	Yes, tested with 30 participants to evaluate usability and performance.
[12]	Indoor Navigation for the Blind	Obstacle avoidance algorithms, spatiotemporal trajectory prediction model.	Trajectory prediction	Yes, experimental evaluation of the trajectory model in a multi-floor mall.
[3]	Evaluation of ETAs with Parrot-VR and Audomni	Audomni, Parrot-VR, various sensors.	VR-based evaluation of navigation tools	Yes, 19 BLV participants tested navigation in large urban environments using Audomni.
[5]	LidSonic V2.0	LidSonic V2.0, LiDAR with servo motor, ultrasonic sensor, Arduino Uno.	Deep learning for object classification	Yes, prototype tested in real-world environments to detect and classify obstacles.
[58]	Voice Navigation System	Computer vision, deep learning, GPS, smartphone-based platform.	Real-time obstacle recognition and classification	Yes, experimental tests with users in various environments to guide navigation.
[59]	Navigation Aid Using Spatialized Sound	Inertial sensors, GPS, camera, deep learning.	Neural networks for spatialized navigation	Yes, real-time navigation tasks using 3D spatialized sound and obstacle feedback.
[60]	Smart Cane with IoT and Image Processing	Camera, ultrasonic sensor, image processing, IoT.	Obstacle detection through image analysis	No
[61]	Architecture for Autonomous Navigation for the Visually Impaired	ADAS, supercomputing, artificial intelligence.	Advanced Driver Assistance for spatial navigation	No
[62]	EyeCane	EyeCane, 2-meter obstacle detection sensor.	Obstacle detection and navigation	Yes, users performed obstacle detection tasks in a real-world environment.

**Table 7:** Systems with artificial intelligence for the navigation of visually impaired people.

Citation	Main Topic	Technology Used	Algorithm/Method Used	Does the System Have an Experimental Phase?
[63]	Blind Navigation with YOLO-SLAM	YOLO for object detection, SLAM for simultaneous localization and mapping, human–machine interface.	Combination of YOLO for object detection and SLAM for localization and mapping.	Yes, evaluated recent studies, focusing on test sessions, sensor optimization, and interface improvements.
[64]	Collaborative Route Planning for Guide Dog Robot	Low-speed guide dog robot (GDR), energy optimization, virtual–real collaborative scenarios.	Energy consumption integral equation to optimize navigation efficiency.	Yes, experiments showed energy savings of 6.91% in straight motion and 10.60% in curved motion.
